# Topoisomerases as Targets for Novel Drug Discovery

**DOI:** 10.3390/ph18111693

**Published:** 2025-11-07

**Authors:** Andrej Perdih

**Affiliations:** 1 National Institute of Chemistry, Hajdrihova 19, SI 1000 Ljubljana, Slovenia; andrej.perdih@ki.si; Tel.: +386-1-4760-376; 2 Faculty of Pharmacy, University of Ljubljana, Aškerčeva 7, SI 1000 Ljubljana, Slovenia

DNA topoisomerases (topo) are essential enzymes that maintain the integrity of the genome by regulating the topological state of DNA during replication, transcription, recombination and repair [1]. These enzymes, which are found in bacteria, archaea, eukaryotes and even some viruses, are categorized into type I and type II topoisomerases, which are distinguished by their mechanisms of transient single- or double-strand DNA cleavage and strand passage that allows DNA topology to be altered [2]. Due to their unique ability to manipulate DNA topology, topoisomerases are often referred to as the “Magicians of the DNA world”, as “in their presence, DNA strands or double helices can pass through each other as if all physical boundaries had disappeared” [2].

The first member of this protein family, the protein ω, now known as *Escherichia coli* topo I, was discovered by James Wang and published in 1971 [3]. This enzyme became the founding member of the type I topoisomerase family and marked the beginning of a rapidly growing field of research. Since then, numerous members have been identified and characterized, leading to vibrant research activity over the past five decades [4].

The ability to manipulate DNA topology has made topoisomerases key targets for antibiotic and cancer therapy, and many drugs acting on them have a long history of success in clinical practice. In the field of infectious diseases, nalidixic acid, introduced in the 1960s, was the starting point for the development of fluoroquinolones, such as ciprofloxacin and levofloxacin, and newer agents such as moxifloxacin, which target bacterial DNA gyrase and topoisomerase IV. In the field of chemotherapeutics, etoposide and teniposide, semi-synthetic derivatives of the natural product podophyllotoxin, were introduced in the 1980s. They inhibit human topoisomerase II by stabilizing the transient covalent complex between topo II and DNA. Anthracyclines, including doxorubicin, daunorubicin and epirubicin, are another established group with a similar mode of action on topo II. Camptothecin, a natural product of *Camptotheca acuminata* discovered in the 1960s, led to the development of the clinically approved topo I inhibitors topotecan and irinotecan in the 1990s, which are used to treat various types of cancer [5].

Despite decades of clinical use, these topoisomerase-targeting agents have several limitations, including antibiotic resistance, off-target toxicity, induction of secondary malignancies, cardiotoxicity and limited tumor selectivity [5,6]. This Special Issue presents a collection of review and research articles that highlight recent progress in overcoming these challenges through the development of new agents targeting these established targets as well as novel insights into the management of chemotherapy-induced toxicities.

A large portion of the review articles highlight the strategies currently being developed for novel topoisomerase inhibitors in oncology and infectious diseases. One of these provides a comprehensive perspective on topoisomerase inhibitors in cancer, covering both topo I and topo II agents, and highlights novel chemical scaffolds, hybrid molecules and clinical candidates that could overcome resistance and toxicity, supported by structural insights for rational drug design [7]. Complementing this, another review looks at the development of bacterial gyrase inhibitors that circumvent fluoroquinolone resistance by targeting either allosteric or ATP-binding sites [8]. The next review focuses on synthetic lethality strategies that combine topoisomerase II inhibition with disruption of DNA repair pathways or oncogenic drivers, offering improved therapeutic selectivity and efficacy [9].

The next section of the Special Issue presents original research contributions. These include 5,8-dimethyl-9*H*-carbazole derivatives with dual topo I/II inhibition, which illustrates the potential of multitarget strategies [10]. The phenotypically discovered thiocarbohydrazone compound, which acts as a catalytic topo II inhibitor, shows cytotoxic activity against cancer cells [11]. Complementing these reports is a newly developed rolling circle amplification assay for rapid, gel-free screening of topo I inhibitors, which enables efficient identification of active compounds [12]. These and other studies in this Special Issue demonstrate how novel chemical scaffolds and innovative screening platforms are helping to advance the next generation of topoisomerase therapeutics.

Overall, the Special Issue *Topoisomerases as Targets for Novel Drug Discovery* demonstrates that successfully tackling these established targets requires translational strategies that combine structural biology [13], medicinal chemistry, computational science [14] and other interdisciplinary approaches to develop next-generation topoisomerase inhibitors that can overcome long-standing clinical challenges.
